# A multimodal precision-prevention approach combining lifestyle intervention with metformin repurposing to prevent cognitive impairment and disability: the MET-FINGER randomised controlled trial protocol

**DOI:** 10.1186/s13195-023-01355-x

**Published:** 2024-01-31

**Authors:** Mariagnese Barbera, Jenni Lehtisalo, Dinithi Perera, Malin Aspö, Mary Cross, Celeste A. De Jager Loots, Emanuela Falaschetti, Naomi Friel, José A. Luchsinger, Hanna Malmberg Gavelin, Markku Peltonen, Geraint Price, Anna Stigsdotter Neely, Charlotta Thunborg, Jaakko Tuomilehto, Francesca Mangialasche, Lefkos Middleton, Tiia Ngandu, Alina Solomon, Miia Kivipelto, on behalf of the MET-FINGER study team, Seliat Adebanke Adeleke, Clara Arvidsson, Isobel Barton, Mehmet Bas, Katrina Cosby, Jennifer Crispin, Lucy Dunn, Margarita Durkina, Ottilia Elebring, Jamie Ford, Parthenia Giannakopoulou, Hanne Gilkes, Hannah Graham, Göran Hagman, Ruby Hall, Helena Hallinder, Arzish Haqqee, Maris Hartmanis, Katri Hemiö, Zuzana Istvánfyová, Dimitra Kafetsouli, Kristina Lakey, Saara Lehtimäki, Lotta Lindström, Peter MacDonald, Aaro Mäkelä, Stefan McGinn-Summers, Carolina Meius, Amnah Mirza, Christine Oesterling, Joanna Ojala, Abdulwarrith Olawale, Isabela Ramanath, Hanna-Maria Roitto, Bilal Sahib, Shonella Singh, Maria Sundell, Shannon Taylor, Devika Tharumaratnam, Kerttu Uusimäki, Johanna Vaarala, Heta Voutilainen, Jessica Åsander

**Affiliations:** 1 Department of Neurology, Institute of Clinical Medicine, University of Eastern Finland, Yliopistonranta 1C, 70211 Kuopio, Finland; 2 The Ageing Epidemiology Research Unit, School of Public Health, Imperial College London, Charing Cross Hospital, St Dunstan’s Road, LondonLondon, W6 8RP UK; 3 Population Health Unit, Finnish Institute for Health and Welfare, Mannerheimintie 166, P.O. Box 30, Helsinki, Finland; 4 FINGERS Brain Health Institute, C/O Stockholms Sjukhem, Box 122 30, SE-102 26 Stockholm, Sweden; 5 Division of Clinical Geriatrics, Center for Alzheimer Research, Department of Neurobiology, Care Sciences and Society, Karolinska Institutet, Karolinska Vägen 37A, 171 64 Solna, Sweden; 6 Theme Inflammation and Aging, Medical Unit Aging, Karolinska University Hospital, Karolinska Vägen 37A, 171 76 Solna, Sweden; 7 Imperial Clinical Trials Unit, School of Public Health, Faculty of Medicine, Imperial College London, Imperial College London, Stadium House, 68 Wood Lane, London, W12 7RH UK; 8 Departments of Medicine and Epidemiology, Columbia University Irving Medical Center, 622 W 168Th St, New York, NY USA; 9 Department of Psychology, Umeå University, 901 87 Umeå, Sweden; 10 Department of Social and Psychological Studies, Karlstad University, 651 88 Karlstad, Sweden; 11 Department of Health, Education and Technology, Luleå University of Technology, 971 87, Luleå, Sweden; 12 Department of Public Health, University of Helsinki, PO BOX 20, 00014 Helsinki, Finland; 13 Diabetes Research Group, King Abdulaziz University, 21589 Jeddah, Saudi Arabia; 14 Directorate of Public Health, Imperial College NHS Healthcare Trust Hospitals, Praed Street, London, W2 1NY UK; 15 Institute of Public Health and Clinical Nutrition, University of Eastern Finland, Yliopistonranta 1C, 70211 Kuopio, Finland

**Keywords:** Alzheimer’s, Dementia prevention, Cognitive impairment, Lifestyle-drug combination therapy, Lifestyle intervention, Metformin, World-Wide FINGERS, Drug repurposing, *APOE*

## Abstract

**Background:**

Combining multimodal lifestyle interventions and disease-modifying drugs (novel or repurposed) could provide novel precision approaches to prevent cognitive impairment. Metformin is a promising candidate in view of the well-established link between type 2 diabetes (T2D) and Alzheimer’s Disease and emerging evidence of its potential neuro-protective effects (e.g. vascular, metabolic, anti-senescence).

MET-FINGER aims to test a FINGER 2.0 multimodal intervention, combining an updated FINGER multidomain lifestyle intervention with metformin, where appropriate, in an *APOE* ε4-enriched population of older adults (60–79 years) at increased risk of dementia.

**Methods:**

MET-FINGER is an international randomised, controlled, parallel-group, phase-IIb proof-of-concept clinical trial, where metformin is included through a trial-within-trial design. 600 participants will be recruited at three sites (UK, Finland, Sweden). Participants at increased risk of dementia based on vascular risk factors and cognitive screening, will be first randomised to the FINGER 2.0 intervention (lifestyle + metformin if eligible; active arm) or to receive regular health advice (control arm). Participants allocated to the FINGER 2.0 intervention group at risk indicators of T2D will be additionally randomised to receive metformin (2000 mg/day or 1000 mg/day) or placebo. The study duration is 2 years. The changes in global cognition (primary outcome, using a Neuropsychological Test Battery), memory, executive function, and processing speed cognitive domains; functional status; lifestyle, vascular, metabolic, and other dementia-related risk factors (secondary outcomes), will be compared between the FINGER 2.0 intervention and the control arm. The feasibility, potential interaction (between-groups differences in healthy lifestyle changes), and disease-modifying effects of the lifestyle-metformin combination will be exploratory outcomes.

The lifestyle intervention is adapted from the original FINGER trial (diet, physical activity, cognitive training, monitoring of cardiovascular/metabolic risk factors, social interaction) to be consistently delivered in three countries. Metformin is administered as Glucophage®XR/SR 500, (500 mg oral tablets). The metformin/placebo treatment will be double blinded.

**Conclusion:**

MET-FINGER is the first trial combining a multimodal lifestyle intervention with a putative repurposed disease-modifying drug for cognitive impairment prevention. Although preliminary, its findings will provide crucial information for innovative precision prevention strategies and form the basis for a larger phase-III trial design and future research in this field.

**Trial registration:**

ClinicalTrials.gov (NCT05109169).

**Supplementary Information:**

The online version contains supplementary material available at 10.1186/s13195-023-01355-x.

## Background

The long preclinical stages of Alzheimer’s disease (AD) preceding cognitive impairment and subsequent dementia provide opportunities for prevention [1]. Despite recent promising findings from anti-amyloid monoclonal antibodies, [2–4] the failure of many single-intervention pharmacological and non-pharmacological trials in AD highlights an urgent need for new multimodal therapy approaches targeting simultaneously several disease mechanisms and modifiable risk factors [5, 6]. AD is a heterogeneous disease, and targeting a single pathology, e.g. amyloid, may not be sufficient to impact disease development and progression to reduce dementia risk [7–9]. The complex pathological processes leading to dementia unfold on multiple levels, e.g. shared risk factors, interaction of different pathology mechanisms, and synergistic effects on cognition. Nonetheless, although multimodal interventions have started to be considered for AD/dementia, only the result of few studies is available to date.

The key challenges in implementing the right interventions for the right people at the right time, and defining accessible and sustainable strategies worldwide, are also clearly emphasized in the recent World Health Organization Guidelines for Risk Reduction of Cognitive Decline and Dementia [10, 11]. Combinations of lifestyle (including multidomain) and pharmacological interventions have been successfully tested in at-risk individuals for the prevention of cardiovascular disease (CVD) [12] and type 2 diabetes (T2D), [13, 14] which share many modifiable risk factors, [15] and potential underlying mechanisms with dementia [9]. Precision approaches combining multimodal lifestyle intervention with repurposed putative disease-modifying-drug could provide effective strategies for dementia prevention or risk reduction.

**Fig. 1:**
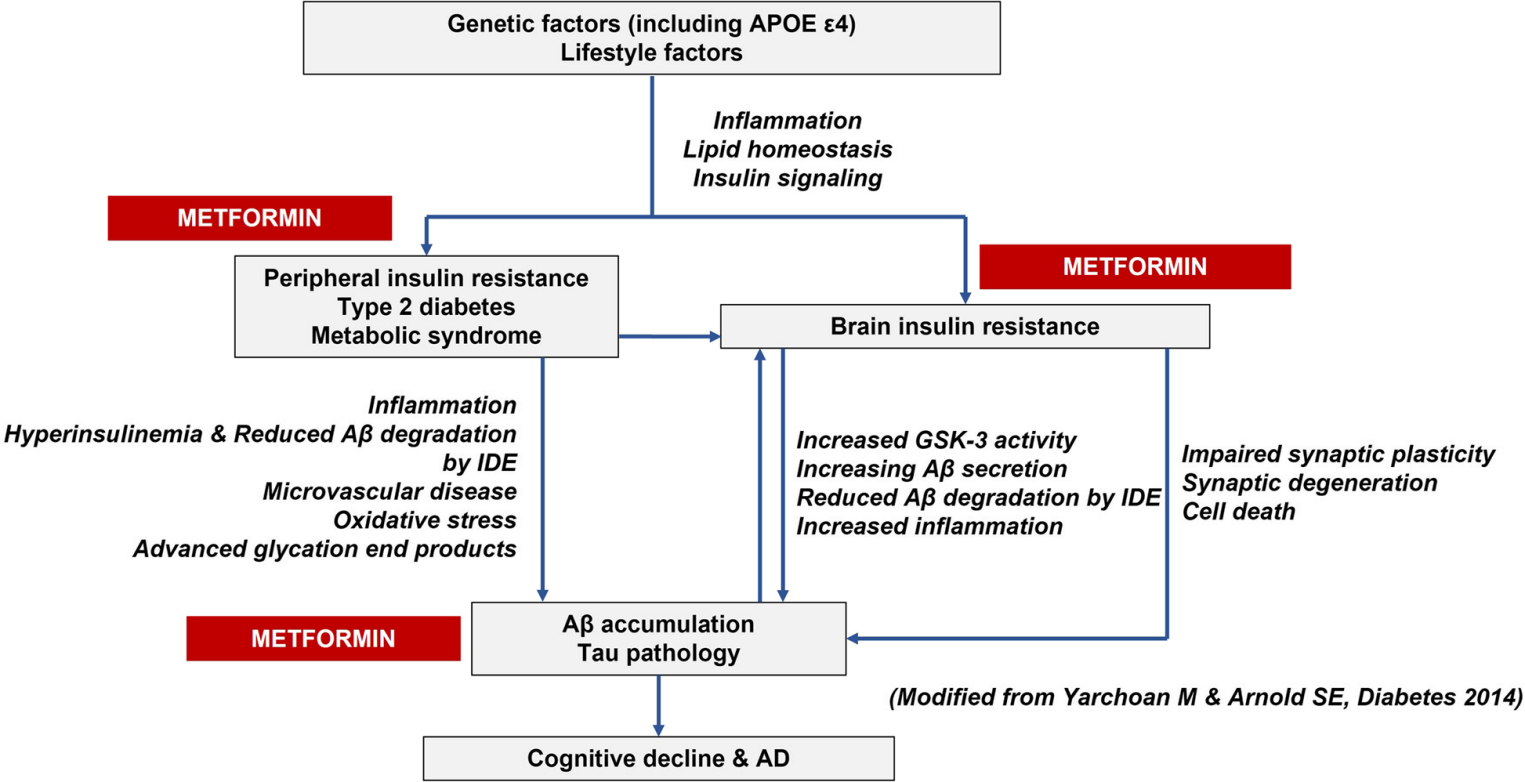
Summary of the mechanisms linking type 2 diabetes (T2D) and its risk factors to dementia/AD and possible metformin targets (Modified from Yarchoan M and Arnold SE, Diabetes, 2014)

So far, the multimodal lifestyle-based Finnish Geriatric Intervention Study to Prevent Cognitive Impairment and Disability (FINGER, ClinicalTrials.gov — NCT01041989) [16] is the only intervention model that showed significant cognitive benefits in cognitively normal at-risk older adults and is now being adapted and tested within the World-Wide FINGERS global network of multimodal dementia prevention trials (to date, 45 + member countries) [17]. Additionally, unhealthy lifestyle habits, T2D, insulin resistance, and increased adiposity (overweight/obesity) have been consistently linked to the risk of dementia/AD through several mechanisms which could be targeted by metformin, the recommended first-line treatment in adults with T2D (Fig. 1) [18–20]. Biological mechanisms of ageing may also play a role in neurodegenerative disorders, and increasing evidence supports a potential role of metformin in counteracting such mechanisms [21]. Furthermore, it has been proposed that the development of novel AD therapeutics could be framed in the context of biological gerontology [22]. This suggests that pharmacologic strategies for decreasing insulin resistance and preventing T2D may also help reduce the risk of cognitive impairment with metformin as a promising candidate for a combined lifestyle-drug intervention to prevent or delay cognitive impairment.

The MET-FINGER study aims to test a FINGER 2.0 multimodal intervention (active arm), combining a Structured Multimodal Lifestyle Intervention (SMLI, based on the original FINGER model) with metformin (where appropriate) in a *APOE* ε4-enriched population of older adults at-risk for dementia, against a Self-Guided Multimodal Intervention (SGMI, general health advice; control arm).

The primary objective is to test the effect of the FINGER 2.0 intervention versus the SGMI on change in global cognition. Secondary objectives are to test the effect of the FINGER 2.0 intervention versus the SGMI on change in individual cognitive domains, functional status, and lifestyle, vascular, metabolic and other dementia-related risk factors. Exploratory objectives will investigate, within the SMLI-metformin combination groups, (i) potential interactions between metformin and lifestyle changes; (ii) potential disease-modifying effects of the lifestyle-metformin combination; and (iii) feasibility of the metformin + lifestyle combination; in the context of prevention of cognitive impairment.

## Methods

### Study design

**Fig. 2:**
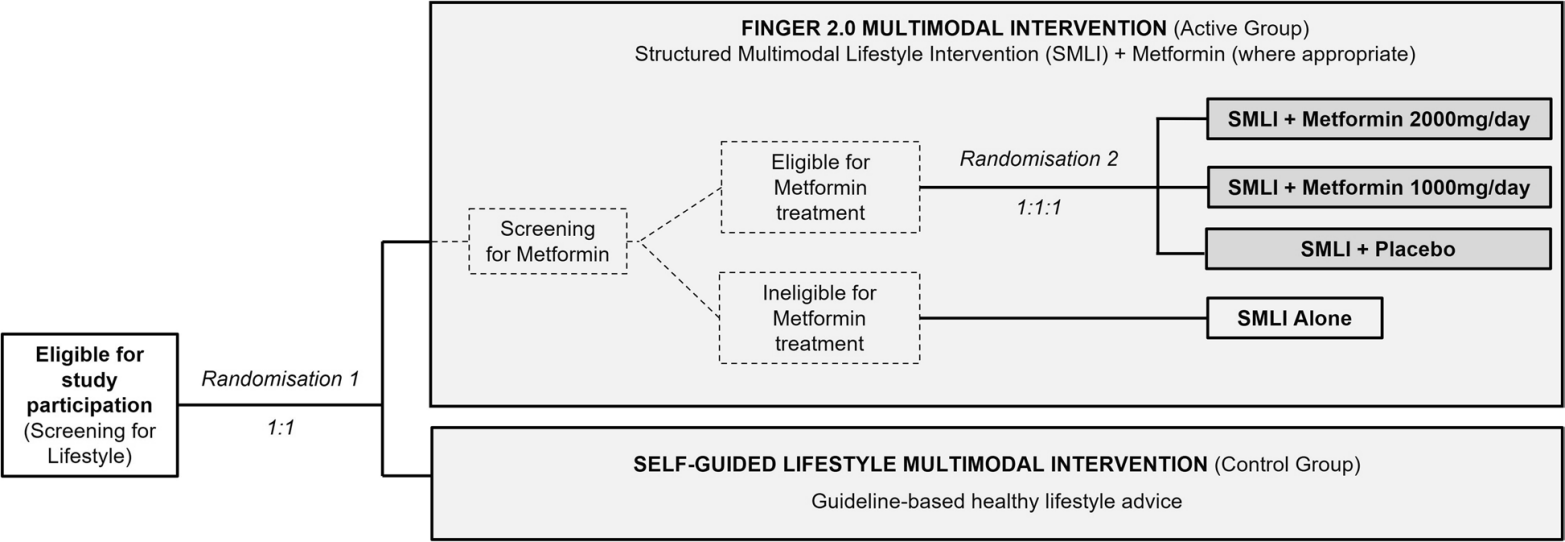
Diagram of the MET-FINGER trial. The main trial comparison is between the FINGER 2.0 Multimodal (combining a Structured Multimodal Lifestyle Intervention (SMLI) and metformin, where appropriate) vs the Self-Guided Multimodal intervention (SGMI). Metformin treatment is included in the FINGER 2.0 Multimodal Intervention through a trial-within-trial where participants who are eligible for metformin treatment will be randomly allocated to one of the three metformin/placebo treatments in addition to the SMLI. Ineligible participants for the metformin treatment will continue participation receiving the SMLI alone. The study, therefore, includes two screening and randomisation steps. The first screening step will assess eligibility for full study participation and eligible participants will be equally randomised to either the FINGER 2.0 or the SGMI. The second screening step will determine specific eligibility to the metformin treatment and eligible participants will be randomised as described above

MET-FINGER is a randomised, controlled, parallel-group, multicentre phase-IIb proof-of-concept clinical trial testing the FINGER 2.0 multimodal lifestyle-based intervention, including a pharmacological trial-within-trial with metformin (Fig. 2). The participants will be first randomised 1:1 to the FINGER 2.0 intervention (SMLI for all participants + metformin where appropriate; active arm) or the SGMI (regular health advice; control arm). Participants allocated to the FINGER 2.0 intervention, and with indicators T2D risk, but no diagnosed/suspected diabetes, will be randomised 1:1:1 to receive metformin 2000 mg/day, metformin 1000 mg/day, or placebo, together with the SMLI. Participants allocated to the FINGER 2.0 intervention but not eligible for metformin treatment will receive the SMLI alone. At each site, both randomisation steps (Fig. 2) are carried out using a computer-generated list from the OpenClinica database. In each randomisation step, the list is stratified by site and programmed using random blocks of various sizes.

The metformin/placebo allocation and treatment will be double-blinded. Primary outcome assessors will be blinded to the FINGER 2.0 versus control allocation, and they will not be involved in the lifestyle intervention.

### Participants

Six hundred participants will be included across three sites to obtain an *APOE* ε4-enriched population (≥ 50%).

In the United Kingdom (UK), participants (*n* ~ 300) will be recruited at Imperial College London (ICL) from the Cognitive Health in Ageing Register: Investigational, Observational and Trial studies (CHARIOT). In Finland, participants (*n* ~ 200) will be recruited via the Finnish Institute for Health and Welfare (THL) biobank. In Sweden, participants (n ~ 100) will be recruited from the electronic database and biobank for clinical dementia research at Karolinska University Hospital (KUH) Theme Inflammation and Aging, Theme medical unit (GEDOC).

Within the relevant registries, potential participants will be first invited for Screening for Lifestyle (Fig. 2) based on age and other eligibility criteria, depending on data availability. Eligible participants will be further invited to participate in the full study. At all sites, participants for whom *APOE*-genotype data is available will be invited for screening starting with an approximate 50/50 ε4 carrier/non-carrier ratio. Carriers have at least one ε4-allele. To ensure 50% minimum enrichment in the overall trial cohort and a similar carrier/non-carrier ratio at all sites, carrier/non-carrier ratio within the eligible population is regularly monitored to adjust the proportion of ε4 carrier/non-carrier invited. The site-stratified randomisation ensures a similar carrier/non-carrier ratio in the two groups at all sites. If needed, participants may be newly genotyped. At Screening for Lifestyle, participants can request disclosure of their APOE genotype. Other recruitment sources (e.g. media campaign) may be used if needed to reach the recruitment target.

Two separate sets of eligibility criteria will be applied within the study (Table 1). The Study Eligibility Criteria aim to identify older adults with some indicators of modifiable risk factors for dementia and cognitive performance at the mean level or slightly lower than expected for age according to population norms, but no substantial cognitive impairment nor conditions preventing safe and effective engagement in the SMLI. The Metformin Eligibility Criteria aim to identify participants with indicators of increased T2D risk, no diagnosed/suspected diabetes and no metformin contraindications. The metformin/treatment will be initiated in all participants who will meet the Metformin Eligibility Criteria (Table 1) and successfully undergo additional metformin-specific safety assessments (e.g. electrocardiography, vitamin B12 blood levels).

**Table 1:** Eligibility criteria

**Study eligibility criteria**
*Inclusion criteria*
1. Age 60–79 years
2. CAIDE Dementia Risk Score ≥ 6 points
3. Cognitive performance at the mean level or slightly lower than expected for age according to local population norms based on the MoCA test and the CERAD verbal learning test. This criterion is met if at least one of the following conditions is met:
a) MoCA score ≤ 25 AND ≥ 15^a^
b) CERAD immediate recall score ≤ 19
c) CERAD delayed recall score ≤ 75% of the last immediate recall attempt
4. Proficiency in the local language (English, Finnish or Swedish)
*Exclusion criteria*^b^
1. Dementia or substantial cognitive impairment (e.g. memory clinic referral needed as judged by the study physician)
2. Current or past use of medications for AD or related diseases (e.g. cholinesterase inhibitors, memantine)
3. Diminished decision-making capacity, not capable of consenting or completing study assessments, based on clinical judgement
4. Other known significant neurologic diseases (including, e.g. Parkinson’s disease, Huntington’s disease, normal pressure hydrocephalus, brain tumour, progressive, supranuclear palsy, seizure disorder, subdural haematoma, multiple sclerosis, or history of significant head trauma with persistent neurologic sequelae or known structural brain abnormalities)
5. Any other condition affecting safe engagement in the intervention (e.g. malignant disease, major depression, symptomatic cardiovascular disease, revascularisation within the previous year)
6. Severe loss of vision, hearing, or communicative ability; conditions preventing cooperation
7. Coincident participation in the active phase of another intervention trial
8. A member of the household already enrolled in the MET-FINGER trial
**Metformin eligibility criteria**
*Inclusion criteria*
1. No diagnosed diabetes or known contraindications to metformin treatment.^c^
2. Elevated adiposity (BMI ≥ 25 kg/m^2^ OR waist circumference > 102 cm in men and > 88 cm in women) OR mildly impaired fasting glucose (6.1–6.9 mmol/l).^d^
*Exclusion criteria*
1. Use of metformin for any indication
2. History of intolerance to metformin used for any indication
3. Diabetes diagnosed or suspected at baseline (e.g. HbA1c ≥ 6.5%^d^, fasting glucose ≥ 7 mmol/l^d^, or 2HPG ≥ 11.1 mmol/l)
4. Hypersensitivity to metformin or to any of the excipients or placebo compounds
5. Metformin contraindications, e.g. hepatic insufficiency and severe renal failure. Individuals with history/presence of known renal or liver disease, congestive heart failure, alcohol abuse, calculated GFR < 60 ml/min^d^ will also be excluded from the study
6. Any type of acute metabolic acidosis (such as lactic acidosis, diabetic ketoacidosis)
7. Acute conditions with the potential to alter renal function such as dehydration, severe infection, and shock
8. Disease which may cause tissue hypoxia (especially acute disease or worsening of chronic disease) such as decompensated heart failure, respiratory failure, recent myocardial infarction, and shock
9. Women of childbearing potential

*2HPG *2-h plasma glucose, *AD *Alzheimer’s disease, *BMI *body mass index, *CAIDE *Cardiovascular Risk Factors, Aging and Dementia, *CERAD *Consortium to Establish a Registry for Alzheimer’s Disease, *GFR* glomerular filtration rate, *HbA1c *haemoglobin A1c, *MoCA *Montreal Cognitive Assessment

^a^MoCA was chosen over MMSE due to its higher sensitivity to subtle and early cognitive changes in older adults without dementia (Markwick, J Clin Exp Neuropsychol, 2012). The cut-offs were chosen to more accurately select a target population that excludes both high performers and people with substantial impairment including dementia

^b^Individually assessed by the study physician. Clinical assessment will provide a more accurate overall picture of the participant’s cognitive and functional level. If the need for memory clinic referral for further evaluation is identified based on clinical judgment, participants will be excluded irrespective of their MoCA or CERAD scores

^c^The process to determine eligibility for Metformin starts after the Randomisation 1 (Fig. 2) and it is structured in specific steps to minimise participant burden. The first step covers eligibility criteria assessed through baseline data (e.g. BMI, waist circumference, fasting glucose, diagnosis of diabetes, and relevant medical history). The second step is an additional screening visit in step-1 eligible participants (remaining eligibility and safety criteria, minus OGTT). Finally, OGTT is conducted in step 2 eligible participants

^d^Assessed with baseline visit data

Dates for the Completion of Enrolment (last participant, Randomisation 1; Fig. 2); Study Completion (last participant last visit); and Primary Analysis (primary publication ready for submission) are currently estimated to be February 2025; February 2027; and September 2029, respectively.

### Data collection

Participants will be invited to a first screening visit, where basic demographics will be collected, and Study Eligibility Criteria (Table 1) will be assessed. Eligible participants will be invited to the baseline visit, where all outcome measures will be assessed prior to the first randomisation step (FINGER 2.0 intervention or SMGI). Participants randomised to the FINGER 2.0 group will start receiving the SMLI and will be further assessed for the Metformin Eligibility Criteria using baseline data and additional assessments at a second screening visit where metformin eligibility assessments will be completed (Table 1). Eligible participants will undergo the second randomisation step, and treatment will be initiated.

All participants will be invited to three additional outcome assessments, where basic safety assessments will be also conducted. At 6 months, a subset of outcomes related to blood sample collection, physical measurements, and health status will be assessed. At 12 and 24 months (end of study) a full outcomes assessment will be conducted, with additional data collection on trial participation adherence and feedback.

Participants randomised to the metformin/placebo treatment will undergo additional safety assessments, every 2 weeks until the full dose is established, and every 3 months after that.

Nominating a study partner to provide information for the assessment of the Clinical Dementia Rating scale Sum of Boxes (CDR-SB) [23] will be supported, but not mandatory.

### Outcomes

Primary and secondary outcomes comparison will be conducted on the FINGER 2.0 multimodal intervention group (active arm) vs the SGMI group (control arm). A comparison of exploratory outcomes will be conducted among the three SMLI + metformin/placebo groups.

Table 2 presents the schedule of the outcomes’ assessment. The bibliography for validated assessment tools, tests, and scales used in the trial is presented in Supplementary Table 1.

**Table 2:** Full schedule of outcomes assessments

Assessments	Outcomes assessment visits			
***Primary and secondary outcomes***	**Baseline**	**6 months**	**12 months**	**24 months**
Neuropsychological Test Battery (Primary outcome and secondary cognitive outcomes)	**X**		**X**	**X**
Clinical Dementia Rating Sum of Boxes (Study Partner required)	**X**		**X**	**X**
Activities of daily living	**X**		**X**	**X**
Healthy Lifestyle Index	**X**		**X**	**X**
Physical measurements (BMI, waist, waist/hip ratio, blood pressure)	**X**	**X**	**X**	**X**
Blood markers (lipid profile and glucose metabolism)	**X**	**X**	**X**	**X**
OGTT (only groups receiving metformin/placebo)	**X**		**X**	**X**
Incident cardiovascular disease	**X**		**X**	**X**
FINGER healthy diet index, nutrient and food intake	**X**		**X**	**X**
Levels of physical activity (self-reported and objectively measured)	**X**		**X**	**X**
Short Physical Performance Battery	**X**			**X**
Hand grip strength, and timed 10-m dual-task test	**X**		**X**	**X**
Center for Epidemiological Studies-Depression Scale	**X**		**X**	**X**
Perceived Stress Scale	**X**		**X**	**X**
Insomnia Severity Index	**X**		**X**	**X**
RAND36 and 15D scales for health-related quality of life	**X**		**X**	**X**
Self-reported data for utilisation of healthcare resources	**X**		**X**	**X**
***Exploratory outcomes***				
Healthy lifestyle changes, e.g. exercise, diet, cardiovascular/metabolic factors and biomarkers, and cognitive activity	**X**		**X**	**X**
AD-related blood biomarkers	**X**	**X**	**X**	**X**
Neuroimaging (Planning phase)	**X**			**X**
Retention rate	Continuously during the study			
Adherence to the SMLI and its individual components	Continuously during the study based on participation to the SMLI structured activities			
Target metformin dose	Continuously during the study with compliance to treatment assessed at each dispensing appointment (every 3 months)			

The bibliography for assessment tools, tests, and scales used in the trial is presented in Supplementary Table 1

*AD* Alzheimer’s disease, *BMI* body mass index, *FINGER* Finnish Geriatric Intervention Study to Prevent Cognitive Impairment and Disability, *OGTT* oral glucose tolerance test, *SMLI* Structured Multimodal Lifestyle Intervention

#### Primary

Change in global cognition measured through a “Composite Neuropsychological Test Battery (NTB) Overall Score” modified from the FINGER trial [24] to ensure the availability of all tests in the trial languages (Table 3) is the trial primary outcome. The 14 test components will be first standardised to their baseline mean and standard deviation. Then, “composite cognitive domain scores” will be calculated by averaging the individual *z*-scores of the components that are relevant for each cognitive domain (memory, executive function, and processing speed). Finally, the composite NTB overall score will be obtained as the average of the composite cognitive domain scores. Composite cognitive domain scores will be calculated if results for at least 3/5 (executive function); 2/3 (processing speed); and 3/6 (memory) tests are available.

**Table 3:** Tests and scores included in the Neuropsychological Test Battery conducted for Primary/Cognitive outcomes assessment

Cognitive test	Item/score included	Relevant cognitive domain
WMSR—Logical Memory	Immediate recall	Memory
WMSR—Logical Memory	Delayed recall	Memory
WMSR—Visual Paired Associates	Immediate recall	Memory
WMSR—Visual Paired Associates	Delayed recall	Memory
HVLT	Learning score	Memory
HVLT	Delayed recall score	Memory
Digit Span	Maximum correct span	Executive functioning
CERAD Category Fluency	Number of correct animals mentioned	Executive functioning
Category Fluency II	Number of correct fruits/vegetables mentioned	Executive functioning
TMT	Score A	Processing speed
TMT	Shifting score B-A	Executive functioning
Stroop	Condition 2	Processing speed
Stroop	Interference score condition 3 -2	Executive functioning
WAIS-DSST	Total score	Processing speed

The bibliography for assessment tools, tests, and scales used in the trial is presented in Supplementary Table 1

*CERAD *Consortium to Establish a Registry for Alzheimer’s Disease, *DSST *Digit-Symbol Substitution Test, *HVLT *Hopkins Verbal Learning Test, *TMT *Trail Making Test, *WAIS *Wechsler Adult Intelligence Scale, *WMSR *Wechsler Memory Scale Revised

#### Secondary

Change in the following secondary endpoints will be included: Composite cognitive domain scores (memory, executive function, processing speed); CDR-SB (only if a study partner is interviewed), and Instrumental Activities of Daily Living; Healthy Lifestyle Index, a composite score based on exercise, diet, lifestyle cardiovascular risk factors, and social and cognitive activity; BMI, waist, waist/hip ratio, blood pressure, lipid profile and glucose metabolism, including OGTT for participants randomised to the metformin/placebo treatment; incident cardiovascular disease; FINGER healthy diet index, nutrients and food intake; Self-reported and objectively measured (with Actigraph) levels of physical activity; Short Physical Performance Battery, hand grip strength, and timed 10-m dual-task test; Center for Epidemiological Studies-Depression Scale; Perceived Stress Scale; Insomnia Severity Index; RAND36 and 15D scales for health-related quality of life; Self-reported data for utilisation of healthcare resources.

#### Exploratory (SMLI + metformin/placebo groups)

(i) Between-group differences in healthy lifestyle changes, e.g. exercise, diet, cardiovascular/metabolic factors and related biomarkers, and cognitive activity
(ii) AD-related blood biomarkers (e.g. amyloid, tau, neurofilament light polypeptide); a neuroimaging sub-study is currently planned
(iii) Retention rate, and adherence to the SMLI, its individual components, and target metformin dose

#### Safety

Liver function (Alanine Aminotransferase, Aspartate Transaminase, Gamma-glutamyl Transferase) and other blood-based markers (e.g. creatinine) will be assessed on all participants at the outcomes assessment. More intensive metformin-specific safety assessments will be conducted on participants randomised to receive the metformin/placebo treatment: Vitamin B12; estimated glomerular filtration rate (eGFR); full blood cell count; vital signs (e.g. blood pressure, heart rate); and medical examination.

#### Other data

Other relevant data will be collected, including, e.g. self-reported demographics (e.g. month and year of birth; age; sex; ethnicity; education;), lifestyle (e.g. alcohol/tobacco consumption; leisure activities), family history of dementia, CVD, and diabetes; relevant medical history; use of concomitant medication and nutritional supplements; self-reported oral health; hearing impairment using the Hearing in Real-Life Environments validated scale and the Digit Triple Test.

### Statistics

The trial primary comparison is Self-Guided versus FINGER 2.0 multimodal intervention arm, and the trial has been powered for this purpose. Power calculations for the primary outcome are based on FINGER data. FINGER included 1260 participants (~ 33% *APOE* ε4 carriers), with an overall drop-out rate of 12% during the 2-year intervention. We estimate that a smaller sample size would be sufficient for MET-FINGER, based on (i) *APOE* ε4 enrichment at least 50% (stratified analysis of FINGER data indicated intervention benefits especially among ε4 carriers); [25] (ii) potential added benefit of metformin; and (iii) optimization of the initial FINGER lifestyle intervention. In FINGER, the observed mean 2-year change (SD) in primary outcome among *APOE* ε4 carriers was 0.10 (0.38) in the control group, and 0.19 (0.34) in the intervention group. A sample size of 506 participants would be needed to demonstrate a between-group difference with 80% power and at 5% significance level. Assuming a 15% drop-out over 2 years, a total sample size of 600 participants would be required.

The primary efficacy analysis will be based on the modified intention-to-treat population, including all randomly assigned participants with baseline and at least one post-baseline observation.

Appropriate transformation to skewed NTB components will be applied. Mixed-effects regression models with maximum likelihood estimation will be applied to analyse change in cognitive scores, as a function of randomization group, time, and group × time interaction. The site will also be included in the model as fixed effect. Non-linearity of change will be taken to account. The other secondary endpoints will be analysed using the appropriate generalised mixed-effects regression model depending on the outcome distribution.

Potential heterogeneity of intervention effects will also be investigated in subgroup analyses, for both primary and secondary outcomes by, e.g. demographics, *APOE* ε4 carrier status, baseline cognitive and functional level, and intervention adherence, although the trial is not powered for such analyses.

For most exploratory outcomes of the lifestyle + metformin trial-within-trial, the same model in the primary analysis of the trial will be used for continuous outcome variables; for categorical outcome variables (e.g. adherence-related outcomes) logistic regression models will be applied. For retention rate and target metformin dose, chi-2 test will be used to compare the three arms.

### Ethical and regulatory considerations

The study will be conducted in accordance with the Declaration of Helsinki’ and the International Conference on Harmonisation for Good Clinical Practice (ICH GCP E6). The study protocol and relevant documents were approved by: the Human Research Authority (HRA, London-Westminster Research Ethics Committee, reference number 22/LO/0053); the Medicines and Healthcare Products Regulatory Agency (MHRA) approval nr. CTA 19174/0429/001–0001 (UK); the Finnish Committee on Medical Research Ethics (Tukija), the Finnish Medicines Agency (Fimea), the Swedish Medical Products Agency (Läkemedelsverket), and the Swedish Ethical Review Authority (EPM), EU-CT Number: 2022–500438-27–01. For full-study enrolment, two separate informed consents will be obtained from each participant, the first for assessing eligibility (Screening for Lifestyle, Fig. 2) and the second for full trial participation (at baseline). If nominated and available, informed consent will be obtained from Study Partners, prior to any data being collected from them.

The trial has been registered at ClinicalTrials.gov (NCT05109169, first release 26th October 2021) and has been adopted onto to National Institute for Health and Care Research – Clinical Research Network portfolio (NIHR-CRN, IRAS ID:1004303).

Potential participants were involved in the study design, including revision of the study material, and are providing continuous feedback during the trial (Supplementary Material SM1).

### Publication, dissemination, and data availability

Any publication reporting data of the present trial will be compiled according to the CONSORT guidelines and checklist. Publications arising from the trial will be available preferably through Open Access. Data arisen from and samples collected during the trial will not be made publicly available but will be available for collaborative sharing, after the main results of the trial have been published, and upon application to and approval by the trial management group. Access is subject to the MET-FINGER legal framework. An access agreement will be prepared and signed by the involved parties.

## Intervention

### FINGER 2.0 multimodal intervention

#### Structured Multimodal Lifestyle Intervention (SMLI)

The SMLI is an updated version of the FINGER intervention designed to provide individually-tailored, recommendations, and support to improve participants’ overall cognitive impairment risk profile, implement healthy lifestyle changes, and promote healthy ageing. It includes five components: *diet*, *physical activity*, *cognitive training*, and *management of cardiovascular/metabolic risk factors* will be delivered through separate structured programmes (Table 4); *social engagement*, will be delivered through group meetings (e.g. for diet, cognitive interventions), structured groups exercise sessions, and by supporting interaction among participants (e.g. independent group exercise).

**Table 4:** Overall summary and schedule of the Structured Multimodal lifestyle Intervention (SMLI) Included in the FINGER 2.0 Multimodal Intervention

SMLI structured programmes^a^	Activity	Study months																							
		**1**	**2**	**3**	**4**	**5**	**6**	**7**	**8**	**9**	**10**	**11**	**12**	**13**	**14**	**15**	**16**	**17**	**18**	**19**	**20**	**21**	**22**	**23**	**24**
**Diet**	Group sessions	X4 (1.5–2 h)						X1 (1.5–2 h)						X2 (1.5–2 h)											
	Individual consultations	X2 (45–60 min)											X1 (45–60 min)												
**Physical activity**	Resistance and balance training (gym)		Once a week; gradually increasing from 30–45 min to 30–60, 45–60, and 60 min (by month 6)																						
	Resistance and balance training (online)			Once a week (duration as above)					Twice per week (duration as above)																
	Independent aerobic exercise			2–4 times per week (30–60 min)					3–5 times week (depending on the number of group training sessions, to have 1 day of rest)																
**Cognitive intervention**	Group sessions				X6 (1–1.5 h)																				
	Independent training						72 sessions (10–15 min); frequency: 3 times a week						Break 3–6 months			72 sessions (10–15 min); frequency: 3 times a week									
**Monitoring of cardiovascular/metabolic risk factors**	Practical consultation			1						1									1						
	Medical consultation			1			1						1												

^a^ The intervention is administered by applying common principles and procedures across sites, but always based on the unique risk profile of individual participants. Additionally, recommendations for diet and management of cardiovascular/metabolic risk factors follow the local/national guidelines, leading to local adaptations and some differences in the way these two components are delivered across sites. Furthermore, when administering the intervention, daily life/habits, needs, living location/conditions and cultural/socio-economic context are considered, both at participant and site level, to ensure tailoring, and support adherence. Continuous communication and participant feedback at 12 months will also help address any potential adherence concerns during the study. Overall, this allows for a highly tailored but still sufficiently harmonised intervention within and across sites. Participants randomised to the SMLI are assigned to a specific Intervention Participation Group (*n* ~ 5–10) and group meetings/exercise session will be organised as much as possible to include the members of a specific Intervention Participation Group. If necessary due to logistics and time availability, participants can attend group meetings/sessions in other Intervention Participation Groups

The *dietary intervention* aims to achieve adequate nutrient intake through a balanced and varied diet. It is based on the local guidelines and the FINGER dietary model, including principles of the Mediterranean diet (higher consumption of fruits/berries and vegetables, fish, vegetable oils and vegetable-fat based margarine, whole grain products and pulses/nuts/seeds, and limited consumption of red meat), and adjusting for country-specific habits and recommendations. These are suitable for the general population and individuals who may have an increased risk of dementia due to, e.g. hypertension, dyslipidaemia, or impaired glucose metabolism. Special emphasis will be put on dietary factors more strongly associated with brain health (e.g. fish, vegetable oils, fruit, vegetables, on a food level; omega-3 fatty acids, folate, vitamin E, and various vitamins from vitamin B group, on a nutrient level). The risk of nutrient deficiencies and excessive alcohol intake will be individually addressed following local guidelines. The intervention will be built on dietary goals, also used to calculate the FINGER Healthy Diet Index as a secondary outcome (Supplementary Table 2).

Trained nutritionists/dietitians will deliver the intervention through individual counselling sessions and group sessions (Table 4). The delivery will be based on theoretical guides (e.g. Intervention Mapping) with emphasis on translating general (e.g. “fish twice a week”, Supplementary Table 2) and/or tailored (e.g. “I will replace breakfast cereal with porridge oats”) goals into concrete behaviours. The “Specific, Measurable, Achievable, Relevant, and Time-Framed” (SMART) [26] principle will be used with the Stages of Change and Social Cognitive theories for goal-setting. Motivational interviewing will be partly applied during the individual meetings, and all goals will be tailored to individual needs and capabilities.

The *physical activity intervention* is based on international guidelines. It includes an individually tailored and progressive resistance and balance training programme (Supplementary Table 3) in group sessions, complemented with independent aerobic exercise (Table 4). Its goal is to make permanent changes in daily physical activity, and it will be administered by a physiotherapist/professional trainer.

The resistance/balance training programme will be delivered in a hybrid on-site/online setting. The on-site/gym sessions will include exercises for the eight main muscle groups (Supplementary Table 3). Online sessions will take place reproducing similar exercises using, e.g. high-resistance elastic bands, and everyday tools normally found at home (e.g. chairs, bottles). For participants to independently control their training and calibrate the load during online sessions, the physiotherapists will guide and instruct them to use the Borg Rating of Perceived Exertion (RPE) scale [27] during the in-person sessions at the gym, based on objective measurements. Adherence to the resistance/balance training programme will be self-recorded by the participant in a specifically designed diary provided by the physiotherapist.

The physiotherapist/professional trainer will plan individual aerobic training programmes, based on each participant’s preferences, needs, and habits. The programme will be regularly revised and adapted as needed and intensity will be adjusted with the Borg RPE scale. Participants will be supported to set up groups to exercise together.

The *cognitive intervention* includes an introductory phase of six group training sessions, alongside, an individual training delivered through a web-based cognitive training programme (Table 4, and Supplementary Material SM2). The *cognitive intervention* includes an introductory phase of six group training sessions, alongside, an individual training delivered through a web-based cognitive training programme (Table 4). The six group meeting sessions are an expansion on the original FINGER cognitive intervention, and their content has been updated to improve motivation and adherence to the individual training through a behaviour change framework [28]. The support programme will provide instructions and guidance for the use of the software-based cognitive training programme, and general education on, e.g. cognition and ageing, risk factors affecting cognition, and strategies to support cognitive functioning. The independent cognitive training will focus on cognitive tasks measuring working memory, executive functions, mental speed, and episodic memory, but no test from the cognitive outcomes NTB is included (Supplementary Material SM2). It consists of a minimum of 144 sessions with an individually adjusted progressive increase in difficulty, delivered three times/week for 10–15 min/session. Two blocks of three tasks respectively are alternated between sessions. The training programme is accessible at home, but training will be offered at the study sites as needed.

The *cardiovascular and metabolic risk factor management intervention* aims to identify the onset of risk factors and support risk reduction. Country-specific evidence-based guidelines will be used at each site. Practical consultations will be conducted at 3, 9, and 18 months, either by a study nurse or a study physician. The consultations will include physical measurements (e.g. blood pressure, BMI) and a review of previous assessments, on which individually tailored feedback will be provided. Clinical/medical consultations will be organised at 3, 6, and 12 months with a study physician. Information on the management of dyslipidaemia; hypertension and diabetes; smoking; alcohol use; weight; as well as other health-related issues that influence the management of vascular risk factors will be provided. Participants will be referred to their primary healthcare provider (according to local healthcare settings) if the need for initiation or adjustment of pharmacological treatment is identified. No medications will be initiated as part of the trial, other than the tested metformin/placebo.

As needed, participants are supported to set and monitor goals to improve their risk profile and will be motivated to adhere to both the relevant lifestyle changes and pharmacological treatment they might have been prescribed by their treating physician.

#### Metformin/placebo treatment

The study-drug treatment will be conducted double-blinded. All participants receiving the SMLI (*n* ~ 300) who are eligible for metformin treatment (Table 1; estimated rate based on data from recruitment sources = 50%, unpublished) will be randomised to either: metformin 2000 mg/day, metformin 1000 mg/day, or placebo.

Metformin will be administered as Glucophage®XR 500 (“SR” in the UK), in form of 500 mg oral tablets. The placebo is prepared based on Glucophage® SR/XR specifications. The placebo will be manufactured to be identical to the active tablets. Both metformin and placebo will be manufactured in bulk by Merck KGaA (Darmstadt, Germany) and prepared at Guy’s and St Thomas’ NHS Foundation Trust Pharmacy Manufacturing Unit (London, UK) in identical dispensing packs, containing sufficient tablets for 3 months. To obtain the target dose, a blinded system will be in place to allow participants to take four tablets/day as follows:

- 2000 mg/day 4 × Glucophage® SR/XR 500
- 1000 mg/day 2 × Glucophage® SR/XR 500 + 2 × placebo
- Placebo 4 × placebo

Participants are recommended to take all four daily tablets together with the evening meal. Gastrointestinal disorders are the most common adverse events; the extended-release formulation will be used to decrease such risks. Additionally, since these undesirable effects occur most frequently during therapy initiation, resolve spontaneously in most cases, and can be reduced by gradual dose increase, the study drug will be titrated from one tablet/day adding one tablet/day each week until the target dose is reached. If the target dose cannot be achieved, participants will be first instructed to reduce the dose to two tablets/day (half-dose) and if the half-dose cannot be achieved, the study-drug treatment will be discontinued.

Based on the safety profile of metformin, the treatment will be discontinued if it is deemed unsafe for the participant due to an adverse event/serious adverse event associated with the treatment (e.g. anaphylaxis, vitamin B12 deficiency); any severe adverse event considered to be related to the study treatment is reported; signs of hypersensitivity are reported; the participant develops diabetes; eGFR < 60 mL/min.

Temporary interruptions will be implemented prior to elective major surgery or other medical procedures as required by the treating physician (e.g. CT scan with contrast infusion), and will be reinstated only after normal renal function is reported. Regardless of the final dose achieved or treatment discontinuation, participants will continue receiving the SMLI. A pre-defined procedure for treatment allocation unblinding will be conducted by an authorised study physician, if required for medical reasons and to ensure participant safety.

Adherence to the study-drug treatment is monitored at each dispensing appointment (i.e. every 3 months) by cross-checking the number of tablets left from the previous dispensing against the number of tablets that should have been left if the participant had fully adhered to the treatment. The assessor will also examine the study-drug diary that participants receive at each dispensing appointment, which includes detailed instructions for the tablets to be taken daily, and allows to record the actual number of tablets taken.

#### Self-Guided Multimodal Lifestyle Intervention (SGMI)

During the outcomes assessment visits (baseline, 6, 12 and 24 months), the control group will receive general, non-tailored, health advice to independently implement healthy lifestyle changes as part of their daily routine. Like in the SMLI, participants in this group will receive the results and explanation of their blood tests; referral/advice to seek medical care will be also provided, if needed. After Randomisation 1 (Fig. 2) is completed, and regardless of group allocation, all participants will receive three educational leaflets providing generic recommendations related to *diet/nutrition*, *physical activity*, and *cardiovascular/metabolic risk factors for dementia*, with content based on local/national guidelines.

## Discussion

MET-FINGER will test, for the first time, a combination therapy of a multimodal lifestyle intervention with a repurposed putative disease-modifying drug, comprising of an updated FINGER 2.0 multimodal intervention model and metformin.

In the context of prevention, the combination of lifestyle intervention and pharmacologic treatment (including metformin) has already showed important advantages when targeting risk factors for CVD and/or T2D such as hypertension, [12] hypercholesterolemia [29] and obesity [30]. Healthy lifestyle changes and pharmacological treatment could have synergistic effects by targeting different biological mechanisms [29] and provide more tailored approaches for disease prevention.

The value of the multimodal lifestyle FINGER model has been demonstrated in terms of prevention potential on cognitive impairment [16]. Evidence of its efficacy has been reported on cognition, functioning, and several related outcomes among at-risk older adults, compared with regular health advice [16, 25, 31–35]. The FINGER intervention was also shown to be feasible and safe [16]. In MET-FINGER, the SMLI component of the FINGER 2.0 intervention has been specifically designed as an upgraded version where intervention tailoring and delivery will be optimised based on the FINGER experience.

A personalised approach is first applied through the highly tailored FINGER multimodal lifestyle model, which is designed to focus on the unique clinical, biological, and lifestyle risk profile of individuals, and to be adaptable to participants’ daily habits and needs. Furthermore, the enrichment of the trial cohort with participants who clearly benefitted more from the FINGER intervention, based on genetic risk (i.e. *APOE* ε4 carriers vs non-carriers), [25] and the addition of a potential disease-modifying drug, based on biological and medical risk stratification, allows, in MET-FINGER, to further refine the precision prevention strategy. This is in line with a definition of “precision prevention”, which entails tailoring risk reduction to a wide range of AD- and dementia-related factors [36].

The choice of metformin as a plausible and promising pharmacological treatment to combine with the SMLI was based on the well-established link between T2D and dementia/AD, and evidence of its relevance for AD/dementia prevention [18–20]. Metformin crosses the blood–brain barrier in animal models as well as humans, [37] activates AMPK in CNS tissue; ameliorates brain insulin resistance; and reduces AD pathology in preclinical models [18]. It showed beneficial effects on several risk factors for dementia/AD, e.g. peripheral insulin resistance, T2D, metabolic syndrome, elevated adiposity, and cardiovascular factors [19]. Metformin was also shown to counteract mechanisms of senescence, [38–40] which is increased in AD and other neurodegenerative disorders, [21] independently of its anti-diabetic action [41]. A beneficial association between metformin (compared with sulfonylureas or non-users) and dementia risk was also reported in large observational studies [42, 43]. Recently, a large (*n* = 210,000 +) cohort study reported that, in patients with T2D, metformin treatment was significantly associated with lower dementia risk compared with no treatment [44]. Only few RCTs (in individuals with MCI) have so far been conducted testing metformin in the context of dementia/AD with promising results on cognition and neuroimaging, [37, 45] and one RCT is currently ongoing (ClinicalTrials.gov NCT04098666), but the combination of metformin and healthy lifestyle changes has never been tested for dementia/AD prevention.

Metformin is a safe, widely available and affordable drug and the first-line treatment in adults with T2D [46–48]. In older adults, metformin appears better than, or at least as safe and effective as, other available treatments for T2D, with the main safety concerns in this age group being gastrointestinal intolerance, and vitamin B12 depletion [49]. To minimise the risk of gastrointestinal symptoms, the modified-release formulation was preferred, and a weekly titration of the study-drug dose will be implemented. Metformin may also lower the vitamin B12 level in some people, [50, 51] which can also have detrimental effects on cognition [52]. Blood levels of vitamin B12 will be assessed twice a year during the study, and effective monitoring of participants’ health status will be ensured by additional safety assessments on the groups receiving the study drug, with a more intensive frequency during the titration period (e.g. creatinine/eGFR for renal function, vital signs, targeted physical examinations).

Two doses (1000 mg/day and 2000 mg/day) of metformin or placebo will be tested in combination with the SMLI. The maximum dose in this trial is also the maximum approved dose for use in people with T2D in many countries. The rationale for the dosage choice was carefully considered. The study drug is authorized in the UK for use in people with pre-diabetes, including older adults, provided that regular assessment of renal function is performed. In MET-FINGER, metformin/placebo treatment will be restricted to non-diabetic people with risk factors for T2D. Several trials have successfully tested the efficacy and safety of metformin to prevent T2D in at-risk people [53]. In particular, the largest (*n* = 3234) study of this kind [54] reported treatment-beneficial effects both at the end of the primary study (mean follow-up = 2.8 years), as well as after 15-year follow-up [55]. No cases of lactic acidosis (the most serious adverse event reported for metformin) were observed in over 15,000 person-years of exposure to metformin, confirming the results of a comprehensive systematic review on the metformin-related risk of lactic acidosis. Doses up to 2000 mg/day were also shown to be safe in other repurposing trials for non-diabetic indications.

## Limitations

The main limitation of this study design is that, although it will provide exploratory data, it cannot provide efficacy data on the added benefits of metformin compared with lifestyle alone. However, several key factors were considered when this phase-IIb proof-of-concept was preferred to a larger phase-III efficacy trial design. First, there is uncertainty regarding the most suitable metformin dose when combined with lifestyle in older individuals at risk of dementia. In a previous RCT (*n* = 80), only 10% of participants tolerated the standard 2000 mg/day dose of immediate-release metformin, mostly due to gastrointestinal discomfort [45]. Additionally, there is limited evidence on interactions between metformin and lifestyle approaches (e.g. diet, exercise), [56] and it is unclear what dose of metformin maximises the health benefits of the combination with lifestyle changes [57]. There may be differences between healthy volunteers and individuals with T2D or insulin resistance, in younger vs older age groups, and with different types and levels of intensity of exercise. Furthermore, potential added cognitive benefits of the metformin-lifestyle combination are difficult to estimate without any prior studies investigating such effects and more robust evidence is required for power calculations and planning of larger phase-III trials. Additionally, the trial will benefit from the use of well-established and available registers/databases within the three countries as recruitment sources. This readiness cohort approach will ease recruitment, enable effective pre-screening, and allow the link of trial data with the observational parent cohorts. The careful selection of the trial outcomes based on the FINGER design and the WW-FINGERS prospective harmonisation framework, will provide crucial opportunities for joint analysis with other RCTs providing the opportunities for more robust and generalisable evidence.

Overall, findings from MET-FINGER and other ongoing placebo-controlled metformin RCT in MCI (NCT04098666) will inform decisions on future phase III precision prevention trials, including, e.g. selection criteria (both metformin eligibility criteria and criteria related to increased lifestyle-based and/or genetic dementia risk); metformin dose selection for combination with lifestyle intervention; and overall trial design, based on the target at-risk population and evidence-based estimates for power calculations, e.g. 3- or 4-arm design [58].

## Conclusions

In conclusion, this trial bridges the gap between pharmacological and non-pharmacological interventions for dementia prevention by proposing a novel precision prevention approach. MET-FINGER will address multiple risk factors and disease mechanisms simultaneously, while considering that their contributions to the overall dementia risk may have a different weight in each individual. The FINGER 2.0 intervention will include person-specific adjustments, and metformin will be given only to people who are most likely to benefit from it, in a pragmatic approach close to a real-life scenario where disease-modifying drugs can achieve optimal effects only in specific at-risk individuals. Findings from this trial may provide invaluable evidence for the development, design and refinement of a novel line of dementia prevention. Such strategies will be based on implementing the most effective solution, targeted to individuals who may benefit most, at the most appropriate time. In the future, MET-FINGER may also be used as a model for novel lifestyle intervention-pharmacological treatment combination approaches where other repurposed or newly developed AD drugs could be tested.

### Supplementary Information

**Additional file 1:** **SM1.** Public and Participant Involvement. Description of the contribution of potential participants in the study design. **SM2.** Cognitive Training Programme – Detailed description. Detailed description of the web-based cognitive training programme. **Supplementary Table 1.** Bibliography for assessment tools, tests, and scales used in the trial. **Supplementary Table 2.** Pre-defined goals of the diet intervention programme. **Supplementary Table 3.** Progression of the resistance and aerobic training programmes.

## Data Availability

Data arisen from and samples collected during the trial will not be made publicly available but will be available for collaborative sharing, after the main results of the trial have been published, and upon application to and approval by the trial management group.
