# Supplementary material for: A multimodal precision-prevention approach combining lifestyle intervention with metformin repurposing to prevent cognitive impairment and disability: the MET-FINGER randomised controlled trial protocol
Source: Alzheimers Res Ther. 2024 Jan 31;16:23. doi: 10.1186/s13195-023-01355-x (PMC10829308; doi:10.1186/s13195-023-01355-x)
Supplement: Supplementary file 1 — Additional file 1: SM1. Public and Participant Involvement. Description of the contribution of potential participants in the study design. SM2. Cognitive Training Programme – Detailed description. Detailed description of the web-based cognitive training programme. Supplementary Table 1. Bibliography for assessment tools, tests, and scales used in the trial. Supplementary Table 2. Pre-defined goals of the diet intervention programme. Supplementary Table 3. Progression of the resistance and aerobic training programmes. [file 13195_2023_1355_MOESM1_ESM.docx]

**Barbera et al.**

**A multimodal precision-prevention approach combining lifestyle intervention with metformin repurposing to prevent cognitive impairment and disability: the MET-FINGER randomised controlled trial protocol.**

**SUPPLEMENTARY MATERIAL**

**SM1. Public and Participant Involvement**

The MET-FINGER protocol was developed with the involvement of older-adult members of the public who, based on age, may qualify for the invitation to the MET-FINGER trial or have already participated in previous multidomain trials aimed at prevention of dementia and/or cognitive decline.

In the UK, a panel meeting/focus group was conducted in December 2020 including ten members of the CHARIOT register who met the MET-FINGER age inclusion criterion (60-79 years). Participants were selected to have a mixed representation of different ethnicities and various postcodes across London, and a balance between men and women. The meeting took place via the online platform Zoom and was chaired by an Assistant Psychologist from the local study team. Three members of the MET-FINGER study coordinating team were also present. The pre-defined topic list covered a range of items from recruitment-related participant-facing documents, informed consent forms, study design, as well as the intervention’s structure and delivery. Participant information sheets were revised based on the feedback obtained during this meeting.

In Finland and in Sweden, prospective feedback was sought through qualitative studies carried out within the original FINGER (Kulmala et al., 2018) and the FINGER-based MIND-ADmini trial cohorts (Akenine et al., 2022). The qualitative study within the MIND-ADmini trial also included study partners (Thunborg et al., 2020). Based on opinions, inputs and feedback collected during these two trials, key components of the FINGER multimodal lifestyle intervention were adapted with the aim to improve adherence and involvement, for example through further support of the social component of the intervention. Feedback from the MIND-ADmini trial focused mostly on the adherence to the physical activity intervention and on how to improve approaches more specifically tailored on each participant. New assessment and intervention tools were introduced to this aim, e.g., Borg RPE, which will help participants to assess the most feasible and realist level of exertion in a more independent and tailored manner.

During the trial, continuous feedback on the intervention and its delivery, as well as the general conduction of the trial, will be collected from all participants, both orally, during each meeting and consultation, and in writing, through structured questionnaires administered at the assessment visits.

The Trial Steering Committee also includes lay independent members within the age-range of the MET-FINGER participants.

**Supplementary Material 2. Cognitive Training Programme – Detailed description**

The cognitive training programme is an in-house developed web platform (hosted by Umeå University, Sweden) that supports browsers for computers and mobile devices, such as phones and tablets. The programme includes six cognitive training tasks as shown below in Figure SM2-1. Two running-memory span tasks are used to train updating of single items in working memory (e.g., 4Numbers and 4Marbles). For both tasks, five lists of items of variable list-lengths are randomly presented and the task is to recall the last four presented items in the correct order. Each item is presented for 2 sec/item. To manipulate difficulty level, the list-lengths are varied, where the low level consists of short list-lengths and is considered less cognitively taxing due to less updating. The level of difficulty is adjusted when the participant scores at 80%, that is 16 correct recalled items/positions. If the participant scores 20% or less, a lower level is presented.

In the working memory task (e.g., Tiles), squares are presented one by one at a rate of 2 sec/item in a 4x4 grid. The task is to remember all the presented locations in the correct order. Three trials are presented for each span level. The span level is increased by one item when the participant correctly recalls two out of three trials at the current span level, and otherwise is decreased by one item. Nine trials are presented per task.

Episodic memory is trained with an associates-task where three words are presented, and the participant is asked to bind them together into a coherent image during encoding (Triplets). The three words consist of a cue word and two target words. The cue word always denotes a place/scene such as a library, and the first target word is a concrete object (e.g., a tulip) and the second target word a colour (e.g., green). Twelve word-triplets are shown for 10 sec/triplet and the participant is told to create an interactive image in their mind associating the colour with the object and placing it in the scene (for example, “a giant green tulip growing in the library”). At retrieval, the cue word is presented, e.g., Library, and the subject must recognize among three distractors the object as well as the colour that was associated with the particular scene/place. The level of difficulty is adjusted when the participants score at 80 % correct. Difficulty level is manipulated by presentation time: the task starts with a presentation time of 10 sec/triplet and decreases by two seconds when 80% correct, down to a minimum of 2 sec/triplets and up to a maximum of 12 sec/triplet.

The second episodic memory task is a classical memory game task (Pairs). Here, pairs of different shapes are randomly displayed face down and the task is to find all pairs as fast as possible and with the fewest number of attempts (turn of cards). The number of pairs are increased by one if fewer than two mistakes have been made and decreased by one if more than four mistakes have been made. A “mistake” is defined as the failure to select the corresponding card in a pair, even though it has previously been revealed, i.e., forgetting it. The number of cards presented can vary between six and 24.

An alternating-runs paradigm is used to train mental set shifting, where two tasks are performed (e.g., Shift). In Task A, the participant has to decide whether the digit is odd or even by pressing the left arrow key when odd (1,3,7,9) and the right key when even (2,4,6,8). In Task B, the participant has to decide whether the number presented on the screen is higher or lower than 5, by pressing the left arrow key when lower (1,2,3,4) and the right key when higher (6,7,8,9). The task consists of 192 trials divided up in six blocks of 32 trials each. During each block, a square divided in four areas is displayed on the screen, serving as a cue as to what task to perform. In the two upper areas of the square, Task A is to be performed, and Task B in the two lower areas. The task begins with one block of Task A followed by a block of Task B and trains mental speed. Thereafter, comes four blocks of shifting between Task A and B. This task is not adaptive, and the participant is encouraged to work as fast and correct as possible.

The cognitive training programme is based on cognitive tasks used in our prior research on healthy older and younger adults, persons with stress-related illnesses and Parkinson’s Disease (Dahlin et al., 2008; Malmberg Gavelin et al., 2018; Ngandu et al., 2015; Sandberg et al., 2014; Walton et al., 2021), where results have shown improved performance in trained tasks as well as near transfer effects that are maintained over time.

**
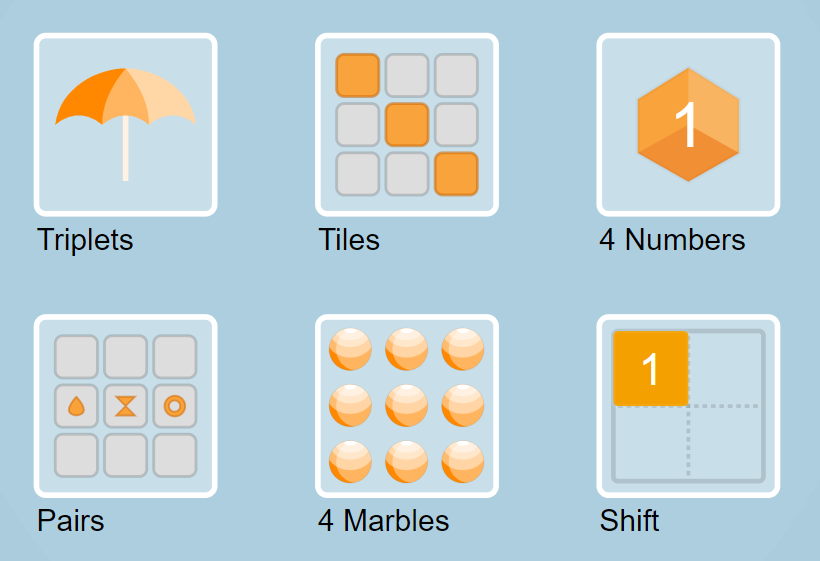
Figure SM2-1**

The six training tasks where participants train by alternating between tasks in row 1 at one session and those in row 2 at the next.

**Supplementary Table 1. Bibliography for assessment tools, tests, and scales used in the trial.**

| **Validated Test/**  **Scale/Assessment Tool** | **Related Assessment** | **Reference** |
| --- | --- | --- |
| MoCA | Cognitive Screening | Nasreddine ZS, Phillips NA, Bedirian V, et al. The Montreal Cognitive Assessment, MoCA: A brief screening tool for mild cognitive impairment. J Am Geriatr Soc. 2005;53(4):695-699. |
| CERAD | Cognitive screening (Word List) and NTB (Category Fluency) | Morris JC, Heyman A, Mohs RC, et al. The consortium to establish a registry for alzheimer's disease (CERAD). part I. clinical and neuropsychological assessment of alzheimer's disease. Neurology. 1989;39(9):1159-1165. |
| WMSR | NTB | Wechsler D. WMS-III —Administration and scoring manual. London, UK: Psychological Corporation Ltd; 1998. |
| HVLT | NTB | Shapiro AM, Benedict RH, Schretlen D, et al. Construct and concurrent validity of the hopkins verbal learning test-revised. Clin Neuropsychol. 1999;13(3):348-358. |
| Digit Span Test | NTB | Lezak MD, Howieson DB, Bigler ED, and Tranell D. Neuropsychological Assessment. 2012. New York: Oxford University Press. |
| TMT | NTB | Reitan RM. Validity of the trail making test as an indicator of organic brain damage. Percept Mot Skills. 1958;8(3):271-276. |
| Stroop | NTB | Golden C. Stroop color and word test: A manual for clinical and experimental uses. Chicago, IL, USA: Skoelting; 1978. |
| WAIS-DSTT | NTB | Wechsler D. Wechsler adult intelligence scale. 3rd ed. New York: The Psychological Corporation; 1997. |
| CDR | Functional status | Morris JC. The clinical dementia rating (CDR): Current version and scoring rules. Neurology. 1993;43(11):2412-2414.  Hughes CP, Berg L, Danziger WL, et al. A new clinical scale for the staging of dementia. Br J Psychiatry. 1982;140:566-572.  Berg L. Clinical dementia rating (CDR). Psychopharmacol Bull. 1988;24(4):637-639. |
| Instrumental Activities of Daily Living | Functional status | Katz S, FordD AB, MoskowitzZ RW, et al. Studies of illness in the aged. the index of adl: A standardized measure of biological and psychosocial function. JAMA. 1963;185:914-919.  Katz S, Akpom CA. A measure of primary sociobiological functions. Int J Health Serv. 1976;6(3):493-508.  Lawton MP, Brody EM. Assessment of older people: Self-maintaining and instrumental activities of daily living. Gerontologist. 1969;9(3):179-186. |
| FINGER Healthy Lifestyle Index | Lifestyle | Barbera M, Lehtisalo J, Rissanen H, et al. Development of a healthy lifestyle index within a multidomain intervention aimed at prevention of cognitive impairment and dementia, and its association with cognition: Results from the FINGER trial. Alzheimers Dement. 2021;17(Suppl.10): e053388. https://doi.org/10.1002/alz.053388. |
| FINGER Healthy Diet Index | Diet | Lehtisalo J, Levälähti E, Linström J, et al. Dietary changes and cognition over 2 years within a multidomain intervention trial-The Finnish Geriatric Intervention Study to Prevent Cognitive Impairment and Disability (FINGER).  Alzheimers Dement. 2019;15(3):410-417. |
| FFQ | Nutrients and food intake | Hollis JL, Craig LC, Whybrow S, et al. Assessing the relative validity of the scottish collaborative group FFQ for measuring dietary intake in adults. Public Health Nutr. 2017;20(3):449-455.  Paalanen L, Mannisto S, Virtanen MJ, et al. Validity of a food frequency questionnaire varied by age and body mass index. J Clin Epidemiol. 2006;59(9):994-1001 |
| Minnesota Questionnaire | Self-reported levels of physical activity | Taylor HL, Jacobs DR,Jr, Schucker B, et al. A questionnaire for the assessment of leisure time physical activities. J Chronic Dis. 1978;31(12):741-755. |
| SPPB | Physical function | Guralnik JM, Simonsick EM, Ferrucci L, et al. A short physical performance battery assessing lower extremity function: Association with self-reported disability and prediction of mortality and nursing home admission. J Gerontol. 1994;49(2):85. |
| CESD | Depression symptoms | Lewinsohn PM, Seeley JR, Roberts RE, et al. Center for epidemiologic studies depression scale (CES-D) as a screening instrument for depression among community-residing older adults. Psychol Aging. 1997;12(2):277-287. |
| PSS | Stress-related symptoms | Cohen S, Kamarck T, Mermelstein R. A global measure of perceived stress. J Health Soc Behav. 1983;24(4):385-396. |
| ISI | Sleep problems | Morin CM. Insomnia: Psychological assessment and management. New York: Guildford Press; 1993:363-377. |
| RAND36 | Health-related quality of life | Hays RD, Sherbourne CD, Mazel RM. The RAND 36-item health survey 1.0. Health Econ. 1993;2(3):217-227. |
| 15D | Health-related quality of life | Sintonen H, Pekurinen M. A fifteen-dimensional measure of healthrelated quality of life (15D) and its applications. In: Walker SR, Rosser RM, eds. Quality of life assessment: Key issues in the 1990s. Dordrecht: Kluwer Academic; 1993:185-195. |
| HERE | Hearing impairment | Heinrich A, Mikkola TM, Polku H, et al. Hearing in real-life environments (HERE): Structure and reliability of a questionnaire on perceived hearing for older adults. Ear Hear. 2019;40(2):368-380. |
| DTT | Hearing impairment | Van den Borre E, Denys S, van Wieringen A, et al. The digit triplet test: A scoping review. Int J Audiol. 2021:1-18. |

CDR: Clinical Dementia Rating; CERAD: Consortium to Establish a Registry for Alzheimer’s Disease; CESD: Center for Epidemiologic Studies Depression; DTT: Digit Triplet Test; FFQ: Food Frequency Questionnaire; FINGER: Finnish Geriatric Intervention Study to Prevent Cognitive Impairment and Disability; HERE: Hearing in Real-Life Environments; HVLT: Hopkins Verbal Learning Test; ISI: Insomnia Severity Index; MoCA: Montreal Cognitive Assessment; PSS: Perceived Stress Scale; SPPB: Short Physical Performance Battery; TMT: Trail Making Test; WAIS-DSTT: Wechsler Adult Intelligence Scale- Digit-Symbol Substitution Test; WMSR: Wechsler Memory Scale Revised.

**Supplementary Table 2. Pre-defined goals of the diet intervention programme.**

| ***At Nutrient Level*** | | |
| --- | --- | --- |
| **Nutrient** | **Goal** | **FINGER HDI** |
| Protein | 15-20 E% | Goal 1 |
| Total fat | 25-40 E% | - |
|  | 2/3 consisting of polyunsaturated and mono-unsaturated fatty acids | - |
| SFA | ≤ 10 E% | Goal 2 |
| PUFA | 5-10 E% | Goal 3 |
| Omega-3 fatty acids | 1 E%, of which DHA 200 mg/day | - |
| Carbohydrates | 45-60 E% | - |
| Refined sugars | ≤ 10 E% | Goal 4 |
| Dietary Fibre | 25-35 g/day | Goal 5 |
| Alcohol | ≤ 5 E% | Goal 6 |
| Salt | ≤ 6 g/day | - |
| ***At Food Level*** | | |
| **Food** |  | |
| Butter/oil | Replacing butter and other SFA sources with vegetable fats/oils | - |
| Fish | ≥ 2 portions (125-150g)/week of fatty fish (e.g., salmon, mackerel) | Goal 7 |
| Fruits and Berries | ≥ 200g/day | Goal 8 |
| Vegetables | ≥ 200g/day | Goal 9 |
| Cereals | Favouring whole grain products | - |
| Dairy | Favouring low-fat options | - |
| Red Meat | ≤ 500g/week | - |
| Sugars | Limiting drinks and food with added sugars | - |
| Alcoholic drinks | ≤ 2units (men) or ≤ 1unit (women) per day | - |

E: Daily Energy Intake; HDI: Healthy Diet Index; PUFA: Polyunsaturated Fatty Acids; SFA: Saturated Fatty Acids.

**Supplementary Table 3. Progression of the resistance and aerobic training programmes.** The resistance training programme will include the eight main muscle groups (knee extension and flexion, abdomen and back muscles, rotation, upper back and arm muscles, and bench press for lower extremity muscles)

|  | **Month 3** | **Month 4-6** | **Month 6-8** | **Month 8-24** |
| --- | --- | --- | --- | --- |
| ***Resistance Exercise*** | | | | |
| *Session frequency/week* | 1-2 | 1-2 | 2 | 2-3 |
| *Duration of exercise, min* | 30- 45 | 30- 60 | 45- 60 | 60 |
| *Number of muscle groups* | 8-10 | 8-10 | 8-10 | 8-10 |
| *Repetitions/set* | 8-15 | 8- 20 | 8- 20 | 8- 20 |
| *Number of sets* | 2 | 2-3 | 1-3 | 2-3 |
| ***Aerobic Exercise*** | | | | |
| *Session frequency/week* | 2 | 2-3 | 3-4 | 3-5 |
| *Duration of exercise, min* | 30-45 | 30-45 | 30-60 | 45-60 |
